# Lack of effective communication between communities and hospitals in Uganda: a qualitative exploration of missing links

**DOI:** 10.1186/1472-6963-9-146

**Published:** 2009-08-12

**Authors:** Elizeus Rutebemberwa, Elizabeth Ekirapa-Kiracho, Olico Okui, Damien Walker, Aloysius Mutebi, George Pariyo

**Affiliations:** 1 Department of Health Policy, Planning and Management, Makerere University School of Public Health, Kampala, Uganda; 2 Johns Hopkins Bloomberg School of Public Health, 615 North Wolfe Street, Baltimore, USA

## Abstract

**Background:**

Community members are stakeholders in hospitals and have a right to participate in the improvement of quality of services rendered to them. Their views are important because they reflect the perspectives of the general public. This study explored how communities that live around hospitals pass on their views to and receive feedback from the hospitals' management and administration.

**Methods:**

The study was conducted in eight hospitals and the communities around them. Four of the hospitals were from three districts from eastern Uganda and another four from two districts from western Uganda. Eight key informant interviews (KIIs) were conducted with medical superintendents of the hospitals. A member from each of three hospital management boards was also interviewed. Eight focus group discussions (FGDs) were conducted with health workers from the hospitals. Another eight FGDs (four with men and four with women) were conducted with communities within a five km radius around the hospitals. Four of the FGDs (two with men and two with women) were done in western Uganda and the other four in eastern Uganda. The focus of the KIIs and FGDs was exploring how hospitals communicated with the communities around them. Analysis was by manifest content analysis.

**Results:**

Whereas health unit management committees were supposed to have community representatives, the representatives never received views from the community nor gave them any feed back from the hospitals. Messages through the mass media like radio were seen to be non specific for action. Views sent through suggestion boxes were seen as individual needs rather than community concerns. Some community members perceived they would be harassed if they complained and had reached a state of resignation preferring instead to endure the problems quietly.

**Conclusion:**

There is still lack of effective communication between the communities and the hospitals that serve them in Uganda. This deprives the communities of the right to participate in the improvement of the services they receive, to assume their position as stakeholders. Various avenues could be instituted including using associations in communities, rapid appraisal methods and community meetings.

## Background

The declaration of Alma-Ata in 1978 in the International Conference on Primary Health Care identified community participation as one of the principles for achieving health care that is acceptable to the users [1]. Effective community participation ensures that the needs and problems of the community are adequately addressed, and the strategies and methods are socially appropriate [2]. One of the ways in which community members participate is by communicating their views in planning, organization, operation and evaluation of services. Views from the communities are very important in the health care system because they reflect the perspectives and priorities of the public [3]. The decision making processes need to be informed by preferences of not only the professionals and the health care managers but also the general public [4].

Community members are stakeholders in the hospitals that serve them and have a right to participate in the improvement of the services they receive. They have an interest in the hospital and are affected by the hospital services [5]. Incorporating community views in health care delivery has been a challenge in many countries across different continents. Health workers sometimes underestimate the people's potential to discuss adequately health issues as shown from studies in Rwanda [6] South Africa [7,8] and Nigeria [9]. Professional and managerial interests dominate the decision making from studies in Britain [10,11] and Canada [3,12]. Communities have not been participating actively particularly in prioritization as shown in studies from Asia [13,14], Mexico [15] and Tanzania [16,17]. In some countries, community representatives were not always supported by the community especially when they were political leaders as was found in Kenya [18] and Uganda [19]. In others, it was the dynamics of implementation that became a challenge, for example taking care of marginalized communities in New Zealand [20] and looking for avenues to collect community views in Colombia [21] and Canada [22] were particularly challenging.

Challenges of involving the community have been identified and community members complain of inadequate information from health care providers [23,24]. There is paucity of information on how these challenges are being addressed by having communities communicate with health facilities around them especially in low income countries. The community needs to be aware of the services available at the health facilities and the challenges that the health facilities encounter in delivering these services. On the other hand the health facilities need to know what services the community needs, as well as the perceptions of the community about the services provided. Effective communication would assist not only in focussing the services to meet community needs but also in ensuring that they address community concerns. In Uganda, health unit management committees were supposed to link the communities and the health facilities [25,26]. The aim of the study was to explore how communities that live around and utilize the hospital services in Uganda pass on their views to and receive feedback from the management of the hospitals in order to identify areas where communication could be improved.

## Methods

### Study areas and population

The study was conducted in and around eight hospitals from August to December 2007. Four of the hospitals were from three districts from eastern Uganda and the other four from two districts from western Uganda. There were four government owned hospitals and four private-not-for-profit (PNFP) hospitals distributed equally between the two regions. We used hospitals because they are usually the sole providers of secondary care within the districts and hence most of the people who in a way needed secondary care would have to relate to the hospitals. For outpatient conditions in rural areas, the main health providers are drug shops and private clinics and sometimes the lower level health facilities [27-29]. All the hospitals are located in rural areas and act as referral facilities for the lower level health facilities in those districts. A member of the health unit management committee (HUMC) – also called the hospital management board – close to the hospital premises and the administrative head of the hospital (medical superintendent) were targeted for interviews. Names and contacts for the members of the HUMC were received from the medical superintendents. The first option to interview was the Chairperson. In the event of the Chairperson not being available, it was the member residing closest to the hospital. Such a member of the HUMC was selected so as to get a person who is in close contact with both the hospital and the community around it. The HUMCs for government hospitals were constituted by the district local governments. The HUMCs for the PNFP hospitals were constituted by the foundation bodies which were mainly the religious bodies. The main purpose of the HUMCs is to govern the hospital on behalf of the foundation body and some of the members are supposed to be representatives of the community the hospital serves. Focus group discussions were conducted with women and men from the communities that lived within 5 km radius around the hospitals. The community members should have spent at least a year living in the community so that they would have had a chance of getting in contact with the hospitals either as patients or caretakers or be aware of some sick people that would have gone to the hospitals. They were expected to be information rich about the community and the hospital [30] and be conversant with the context of the particular hospital services and how the people communicate with the hospital administration [31].

### Data collection

The data collection methods included eleven key informant interviews (KIIs) (eight with Medical Superintendents of the hospitals and three with members of the HUMCs). These were selected because they would give a broad view of the hospitals interaction with the communities around them [32]. The KIIs were conducted by the investigators themselves in English. The purpose of conducting the KIIs was to obtain in-depth information on how the hospital management communicates with the communities around them and how the community representatives give feedback to the hospitals. They were conducted at the hospital premises for the case of medical superintendents and at the offices or residences of the members of the health unit management committees. The KIIs focussed on how hospitals received information from the communities and how communities gave information on the hospitals about the latter's delivery of services. All medical superintendents were interviewed but only three HUMC members could be interviewed despite frequent attempts to contact members of other HUMCs at the time data was being collected in the respective hospitals.

Eight FGDs -one in each of the eight hospitals – were conducted with health workers who were mainly nurses and midwives in routine hospital care but who also conduct outreaches like for immunization to the surrounding communities. The Senior Nursing Officer in the hospital was asked to name nurses who would have worked in the hospital for more than a year from different departments. The investigator would then select nurses and midwives for the FGD trying to include those in bedside patient care, out patients departments and outreaches. FGDs with health workers were done because health workers are the providers of care and it was deemed important to explore whether there was communication between providers and consumers of care. All the FGDs were conducted in the hospital premises in a secured room. The FGDs focussed on how community members forwarded their complaints to and received feed back from the health workers. Eight FGDs – four with women and four with men – were conducted in the communities around the hospitals. Women were selected because they use hospital facilities most either as patients or caring for sick relatives. Men were also included because they are involved in the decision making of places where women or children get services from in addition to being patients of these hospitals themselves [33]. Of the four FGDs with women, two were conducted in the western region, and two in the eastern region. Within each region, there was an FGD with women around a PNFP hospital and another around a government hospital. The distribution of the FGDs with men was similar. This was to explore diversity of opinion according to regions, sex or hospital ownership. The mobilization of the community participants was done by the District Health Inspectors who were assisted by the members of the village local councils. These were perceived as not involved in delivery of health care at hospitals and their selection would be less influenced by interests to protect or damage the image of the hospitals. The health inspectors contacted local council chairpersons or women representatives at village level who identified the men or women participants respectively. Each FGD was conducted in a different village. FGDs contained either women alone or men alone as this would give an opportunity especially for women to participate more freely than when they would mix with men. FGDs 'give a voice' to the marginalized and researchers are able to get information from categories of people who have little chance of expressing themselves [34]. All the FGDs in the community were conducted in one of the homes of the participants except one FGD with men where the participants preferred a nearby primary school. The participants focussed on how they received information about health services in the hospitals and how they communicated complaints to the hospitals.

The number of participants per each FGD ranged from eight to twelve as recommended [35,36]. FGDs were used to explore the group dynamics in the shared experiences of the community members [37]. The FGDs were conducted by social scientists with degree qualifications and were experienced in conducting FGDs. They were trained by the investigators with the aid of FGD guides. This enabled them to familiarise themselves with the topics for discussion. FGDs for community members were conducted in the local languages of the area to enable the FGD participants express themselves in the language they knew best. For health workers, FGDs were conducted in English. Each FGD had a moderator and a note taker and was attended by one of the investigators who would also take field notes and record the observations. The entire session would be guided by the moderator and the investigator would ask a few questions for clarification only at the very end of the session so as to minimize the influence of the investigator on the information discussed. After eight FGDs in the community, it became very clear that there were no emerging differences between regions, between men and women or between perceptions towards PNFP or government hospitals. Categories became repetitive and data collection was stopped.

### Data management and analysis

All the KIIs and FGDs were tape recorded after getting participants consent. Through tape recording details of the KIIs and FGDs were obtained with accuracy that would not be got from the field notes or from memory. Tape recording also allowed more eye contact between the moderators and the respondents [38]. The KIIs were transcribed by the investigators. The FGDs from community members were transcribed in the local language and later translated into English by the moderators while the ones with health workers were transcribed directly into English. The transcriptions and translations were cross checked by the investigators. Manifest content analysis was used whereby the meaning units were summarized, then coded and then condensed into categories [39].

### Ethical clearance

The study was approved by the Makerere University School of Public Health Institutional Review Board and the Uganda National Council for Science and Technology. Permission to carry out the study was obtained from the district and the local leaders. Informed consent was obtained from all the participants who were involved in the study. At the beginning of each interview or FGD, the research assistant/investigator would introduce him/herself and the people he/she would have come with after which the participants would introduce themselves. The research assistant/investigator would explain the objectives of the study, the expected benefits especially for the community, assure them of confidentiality and anonymity of the data collected and make it clear that they were free not to contribute when they felt they shouldn't, were free to withdraw any time and no services would be withheld because of their non participation. They would all give their consent verbally and individually. Written consent is shunned by potential participants because they develop a feeling of lack of confidentiality with the information they give to researchers. Secondly, some of the FGD participants from the villages do not know how to read and write and would therefore feel handicapped if they have to give written consent.

## Results

The main findings of the study were: 1) Views from HUMCs were detached from those of the community. Whereas HUMCs were supposed to have representatives from the communities, the HUMCs never received views from the community nor gave to communities any feed back from the hospitals. 2) When some community members used mass media like radio to air out their grievances, their grievances were seen as too general to be addressed by higher authorities above the hospital. 3) It was difficult for the hospital administration to respond to views sent through suggestion boxes because they were more of individual needs rather than community concerns. 4) The community members perceived they would be harassed if they complained and had reached a state of resignation preferring instead to endure the problems quietly.

### 1) Health Unit Management Committees as channels of communication

Hospitals had health unit management committees that interacted with the hospital administration through supervisory visits or management meetings. Supervisory visits from foundation bodies took different forms depending on the foundation body of the hospital in question. It was the opinion of most respondents that the PNFP hospitals had regular supervision while the supervision in government hospitals was often done after long periods and was irregular. The interaction between hospitals and management committees were in this respect weak.

*If you compare government hospitals with other private institutions (the PNFP), you will find that the private institutions have foundation bodies. These foundation bodies have management committees that take the responsibility to see whether people receive the services. That one we know. They meet the staff, train and put in them a sense of liking their work. But in government hospital, you might find that, they haven't received any inspector from the district for the whole year to supervise the hospital. It's the government's responsibility especially those that are concerned like district officials to ensure that their hospital provides good services, instead of sleeping in offices without going down to the facility to see how the situation is*. (FGD Men)

Members of the HUMCs were selected from different interest groups. There were variations depending on the ownership but each HUMC had at least one member from religious groupings, political leaders and village opinion leaders. Members of HUMCs who were interviewed had the view that this was adequate.

*The common man is well represented. There is a district councillor here who is representing the district council and the local councillor, a local councillor I mean is from this sub county. Then on our board we have got chairman LC3, then an elder. I am now called an elder because of my long service to this hospital. Then we have people who represent churches. Our chairman is a Reverend Canon. The Reverend Canon represents the Church of Uganda, then there is somebody representing the Catholic Church, and then there is some body representing the Moslems. In fact those are the major religious sects in this area and therefore the communities of the area, around and beyond the district are well represented*. (HUMC Member Government Hospital)

However, the majority of the FGD participants and the medical superintendents thought that these representatives who have other portfolios represent their other interests and not necessarily those of the community. A case in point was the comment from the PNFP hospital medical superintendent on representation by religious leaders:

*I don't think the common man is represented by these religious leaders unless may be if they attend church. The one who attends church is represented because there is good representation by the religious leaders but there is need for more community representation on the board*. (MS PNFP Hospital)

One of the main arguments of the group that saw inadequate representation by the HUMC members was that these members never came to ask for the community views. Even when the HUMC met the hospital administration, the community was never aware of what they discussed.

### 2) Communication through mass media like radio

One of the main channels used to share the community impressions on the health services was the mass media such as the radio stations. The majority of the FGD participants reported using FM radio stations to pass on their views to the hospital authorities. In some of the radio programs, the clients phone in to express what they felt about the services.

Whereas, radios were being utilized as ways of communicating to the health workers, the majority of the community participants perceived that messages which are given on the radio are sometimes vague and generalised. Radios could not address specific issues which affected the clients.

*Sometimes they talk about the health workers and at least that's good for us. The problem is that they don't talk about individuals. They beat about the bush. They can say that health workers in Uganda are behaving poorly. We want the callers to mention the names of those who behaved badly and the bad things they did*. (FGD Men)

On the other hand, most of the health workers felt that such communication through the radio was ineffective because the clients may give incorrect information and yet the health workers did not have an opportunity to respond. This was shared by the majority of the medical superintendents interviewed.

*Well many times we just hear such things on the radio. They take their complaints to the radio they rarely come to us may be if they came we would give them correct information. Sometimes we hear them on the radio saying ABCD that you go to the hospital and you don't get this, that the hospital is doing this. But they have not come to us. They choose media for reasons well known to them*. (MS Government Hospital)

The majority of the medical superintendents further argued that complaints from the communities sometimes are not substantiated. They come as rumours. At other times, the community picks up a general problem that the hospital is not in position to address but possibly a higher authority like Ministry of Health.

*But sometimes you find that the problem needs attention but has to affect the whole of the system for example they say that they are delayed in Out Patients Department, they wait for some time. When I look at the staffing levels here in the hospital, we have a problem. Our staffing level is at 44%. So some of the problems which can be rectified we address them, the ones that need the attention of the management board, district or ministry we try to talk about them but usually they are about service deliveries, waiting time*. (MS Government hospital)

### 3) Communication through suggestion boxes

All the FGDs and KIIs discussed the suggestion boxes as an avenue of communication between the hospital and the community. Suggestion boxes are fixed in public places in the hospital premises where clients, attendants or even the public could write their complaints or suggestions and deposit them for the attention of the hospital administration. Some of the FGD participants, though few in number, indicated having used suggestion boxes. Most of the medical superintendents and the FGD participants did not find the suggestion boxes helpful. To the medical superintendents, the draw back with suggestion boxes is that the clients will give their personal opinions and not those of the community. It becomes difficult for the hospitals to make changes on such suggestions unless if they are persistent and complimented by other information.

*The other thing may be some times they use the suggestion box. But analysis of what they have forwarded before us, we found tends to be their feelings, they become very personal not really talking about the institution and eventually the suggestion box has not helped because of the way they personalize some of their suggestions. That would be a good agent to us and the community around us*. (MS government hospital)

To the majority of the community members, the suggestion boxes were not helpful because the very people they would talk about are the very ones going to open the suggestion boxes.

*The suggestion box is there but the people we report about in that suggestion box are the same people who open it. They will decide not to respond to the complaint and we cannot tell whether they got the complaint or not*. (FGD Women)

### 4) Perception of fear and harassment

Amidst these challenges, the majority of the community participants were not aware of the procedures to follow in case one is wronged. Many of the participants were not aware of the avenues through which the community could pass their grievances about the services they received from the hospitals.

*We do not know where to report in case something goes wrong. Instead, one dies quietly. Also, when you try to interfere with the health worker, it might result into quarrels and fights. We keep quiet because you cannot quarrel with a health worker when he is the one going to treat you*. (FGD Women)

*Let me tell you we fear even if you have something you are not contented with, you fear to mention it because on another day when you have a patient you will have to go back to the hospital. So when they note you that you reported him/her they will treat you poorly. One time they were even put on radio that the nurses in the hospital do not care about patients. But you remember what happened, they could tell us that you always put us on radios, but what brings you here? They could ask us that, "is this the only hospital?" So even if you have something so long as your patient recovers you just have to keep quite*. (FGD Women)

For the health workers, they thought the community was very happy with the work they were doing when they did not come for services, it was because of lack of supplies like drugs which the health workers had no control over.

*I would say the community is very satisfied with our work but of course also depending on the service you have provided. Many patients choose other facilities because they have been discouraged by many things like stock-outs. Government has not maintained stock for a facility like this one. So our stock according to plans from government is not enough and is consumed much earlier than the expected time. So even when the community is willing to come in, the service is not quality because we do not have enough to give out*. (FGD health workers)

However, there was another section of health workers who thought that the community grumbles but never tells them their complaints. Some of those grumbling were considered ungrateful.

*There are some people who are not grateful even if you give them a service they really can't appreciate. They will go around speaking ill of you whereas you did your best. On the other hand, there are those you give a service and they appreciate. Then there are those health workers who don't perform well. They may be found in a bad mood and the clients they work on will always curse*. (FGD health workers)

The majority of the community participants looked at the health workers as having extra ordinary power, having gone to school and having been given power to save people.

*What my colleague has talked about the issue of talking about them over the radio is very true. Whenever somebody could go to the radio, the health workers would tell us that, "you people you went over the radio and you said that we the health workers are like this, why don't you go to other health centres?" It is us the patients who should bend low for the health workers because it is them who went to school, they know where life is and they are the people God gave the gift of saving people*. (FGD Men)

Despite this loss of hope, there were a few FGD participants who maintained that talking about health workers performance on radio at least makes the public aware of the poor services. Though, most of the FGD participants agreed that talking about poor health services on radio was not making services better.

During the FGDs, it was very clear that the majority of the community participants did not see themselves as partners in health care delivery, neither were they recognized as such. When the clients complained about hospital workers, this was interpreted as a criticism, the health workers would fight back and the politicians would not be of any help.

*We fear complaining about the behaviour of health workers because if you complain about one, he/she can talk to his/her colleagues and they give you over doze and you die. Those health workers tell us that for them they have their certificates. The politicians are only voted into power, so even if you are in power you can't do anything. The health workers say that if you complain about them, they can only be given a transfer to another place since they have their documents. In addition to that, someone you did not educate, I don't know how you start reporting that this person did not treat my patient*. (FGD Men)

Few of the community participants were of the view that when they meet the health workers on a one-to-one basis and become friends with the health workers, that is when they could share their experiences of the hospital with them but this was only for the very few that would be friends of health workers.

## Discussion

**Figure 1:**
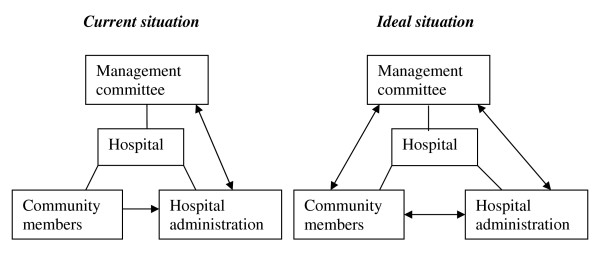
**A comparison of the current links in communication and the ideal situation**.

The available channels have brought about some communication between communities and hospitals but gaps remain. Health unit management committees perceive that they represent the community interests but community members see their interests not catered for. The hospital administrators do not consider complaints given on radio or through suggestion boxes as focussed. Health workers perceive that community members grumble but never come out to speak. There is lack of a direct two-way communication between the communities and management committees or the communities and hospital administration (Figure 1).

Community members are primary stakeholders in hospital service delivery because they are essential to the survival and well being of the organization [40]. They are the hospitals' customers. They need a direct linkage other than through the HUMCs. Health unit management committees did not provide the solution of taking the community views to the hospital or feed back to the community. For both the government owned and PNFP hospitals, the views of the HUMCs were detached from those of the communities around those hospitals. For government hospitals, the situation was worse because supervision was even rare. However, the views expressed by members of HUMCs were considered to be for the interests of the groups they represent like if they were religious groups it would be for religious concerns. Their concerns were not seen as focussed on the health needs of the community. Similar concerns have been raised by other studies. A study from Uganda indicated that some of these representatives are political leaders who may be compromised by political interests, and who may therefore not put the needs of the community first [19]. In a study done in Australia among clients attending a medical clinic, politicians were seen as the least important in championing the priorities of the community [4]. A direct communication between the communities and the hospital administration is needed to enable the interests of the community be received by the hospital without succumbing to intermediate interests of other interest groups.

Hospitals need to know public views but even individual voices need to be heard. Health services are utilized by individuals and each individual will have his or her experience to tell. In an attempt to establish direct links, the community members go on radio or give their messages through suggestion boxes. When the community members expressed their concerns in this way, the management of hospitals considered their concerns as individualized sometimes not congruent with the needs and capacities of the hospitals. Handling individual complaints becomes a challenge for an institution like a hospital. Some studies have noted the constraints that health facilities face and which also determine their capacity to respond to the community needs as noted from United Kingdom [41]. There is need to differentiate between individual and collective forms of patient involvement [42]. If health care delivery programs are to be more effective than they are, communities need to participate as communities beyond individuals [16,17] at the same time cater for individual requirements as individuals need to be seen as "ends in themselves" [43]. Facilities need to get a way of addressing individual complaints because they affect the satisfaction of clients with health services. This in turn may affect the utilization of health services. As individuals, the community members are entitled to contribute to the way their affairs are run much as hospitals need also to get the "public opinion" that sort of gives the voice of the community in general.

The interaction between community members and the health workers should be characterized by the respect of each others' rights. In the face of seemingly "all-too-powerful" health providers, the community members were engrossed in fear and resignation. Whenever comments would come on the radio, community members perceived they were harassed by individual health workers, the very people who would benefit from their comments. It eventually leads to community members not participating in the delivery of their services and this patient non participation has been described also in other studies in England [44]. The relationship between community members and health workers should be guided by the rights of clients to information and the rights of providers to feed back [45]. In this kind of scenario where the community has no voice, providers are not held accountable. In the long route of accountability, health providers are held accountable by politicians, who are voted in by the community in the hope that services will improve. When this long route works, the community can complain to the politicians who are better positioned to present the complaints to the health providers and to demand for action that can remedy the situation [46]. The community did not feel any protection from the politicians. They felt they had nobody to appeal to.

For community members to give and receive communication from the hospitals that serve them there need to be channels through which community members could pass on their views to the hospital administration. It should link up the hospital administration with the community directly. When this short route fails, the long route of accountability should be functional. There needs to be effective communication between the community members and the HUMCs (Figure 1). These communication channels would harmonise individual and community concerns as well as community needs and the hospital capacity to address them. By so doing, clients who use the hospital would have the hope that the quality of the services at the hospitals can be influenced by their views.

There have been attempts to bridge the community – health facility gap. In Colombia associations and consumer service officers were being used to get community views [21] and in Canada citizens juries were being involved [12]. Rapid appraisal methods have been recommended [47] and successful examples of getting community interests using them were conducted in the United Kingdom [48]. Other attempts have been community meetings in Tanzania [49,50]. Another way could be to incorporate individual concerns at planning level [2] so that by implementation stage, the community is already involved. Some community members prefer receiving specified communication messages from family members [51]. These attempts demonstrate that a step can be taken and when complemented by other avenues, an increase in community participation in the planning, organization and control of the health facilities that serve them can be improved.

### Methodological considerations

The qualitative design was used to explore experiences from the community and the hospital management. Using different methods and respondents was aimed at getting an objective view of the data. Though qualitative methods do not give the magnitude and variations across the different categories of the respondents, triangulation across different researcher professions and across the different focus groups and key informants from different regions was very useful to check the consistency and contradictions across and within groups and regions [52,53]. Any disagreement in interpretation was resolved by holding a joint discussion and going back to the original transcripts and contextualizing the meaning units. Having used participants who were purposively selected makes the research findings not representative of the entire population. However the participants were chosen because they were information rich and gave informed opinion about the issue being explored. The number of health unit management committee members interviewed was only three. Their views were not divergent. However, missing out on these interviews with other HUMC members may have unfortunately left out some important information.

## Conclusion

There is lack of effective communication channels between the communities and the hospitals in Uganda. Efforts to individually voice complaints do not get response and the clients perceive they would get abuses from the health workers. This deprives the community members of the rights to participate in the improvement of the health services they get. Various avenues such as regular community surveys, community meetings or user organizations could be instituted to give feedback to the hospitals and to address community needs.

## Competing interests

The authors declare that they have no competing interests.

## Authors' contributions

ER, GP, EEK and OO initiated the concept for the study. ER, EEK, OO and DW developed the tools for the study. ER, EEK and AM participated in the data collection. ER came up with an analysis plan and performed the data analysis. ER and EEK co-wrote drafts of the manuscripts. DW, AM and GWP reviewed and provided substantial inputs into the manuscript. All authors read and approved the final manuscript.

## Pre-publication history

The pre-publication history for this paper can be accessed here:



## References

[B1] Gillam S (2008). Is the declaration of Alma Ata still relevant to primary health care?. BMJ.

[B2] Sule SS (2004). Community participation in health and development. Niger J Med.

[B3] Bruni RA, Laupacis A, Martin DK (2008). Public engagement in setting priorities in health care. Cmaj.

[B4] Wiseman V, Mooney G, Berry G, Tang KC (2003). Involving the general public in priority setting: experiences from Australia. Soc Sci Med.

[B5] Varvasovszky Z, Brugha R (2000). A stakeholder analysis. Health Policy Plan.

[B6] Freyens P, Mbakuliyemo N, Martin M (1993). How do health workers see community participation?. World Health Forum.

[B7] Barker M, Klopper H (2007). Community participation in primary health care projects of the Muldersdrift Health and Development Programme. Curationis.

[B8] El Ansari W, Phillips CJ, Zwi AB (2002). Narrowing the gap between academic professional wisdom and community lay knowledge: perceptions from partnerships. Public Health.

[B9] Uzochukwu BS, Akpala CO, Onwujekwe OE (2004). How do health workers and community members perceive and practice community participation in the Bamako Initiative programme in Nigeria? A case study of Oji River local government area. Soc Sci Med.

[B10] Gorsky M (2008). Community involvement in hospital governance in Britain: evidence from before the National Health Service. Int J Health Serv.

[B11] Callaghan G, Wistow G (2006). Governance and public involvement in the British National Health Service: understanding difficulties and developments. Soc Sci Med.

[B12] Martin DK, Abelson J, Singer PA (2002). Participation in health care priority-setting through the eyes of the participants. J Health Serv Res Policy.

[B13] Murthy RK, Klugman B (2004). Service accountability and community participation in the context of health sector reforms in Asia: implications for sexual and reproductive health services. Health Policy Plan.

[B14] Sepehri A, Pettigrew J (1996). Primary health care, community participation and community-financing: experiences of two middle hill villages in Nepal. Health Policy Plan.

[B15] Zakus JD (1998). Resource dependency and community participation in primary health care. Soc Sci Med.

[B16] Mubyazi GM, Mushi A, Kamugisha M, Massaga J, Mdira KY, Segeja M, Njunwa KJ (2007). Community views on health sector reform and their participation in health priority setting: case of Lushoto and Muheza districts, Tanzania. J Public Health (Oxf).

[B17] Mlozi MR, Shayo EH, Senkoro KP, Mayala BK, Rumisha SF, Mutayoba B, Senkondo E, Maerere A, Mboera LE (2006). Participatory involvement of farming communities and public sectors in determining malaria control strategies in Mvomero District, Tanzania. Tanzan Health Res Bull.

[B18] Kaseje DC, Sempebwa EK, Spencer HC (1987). Community leadership and participation in the Saradidi, Kenya, rural health development programme. Ann Trop Med Parasitol.

[B19] Kapiriri L, Norheim OF, Heggenhougen K (2003). Public participation in health planning and priority setting at the district level in Uganda. Health Policy Plan.

[B20] Boulton A, Simonsen K, Walker T, Cumming J, Cunningham C (2004). Indigenous participation in the 'new' New Zealand health structure. J Health Serv Res Policy.

[B21] Mosquera M, Zapata Y, Lee K, Arango C, Varela A (2001). Strengthening user participation through health sector reform in Colombia: a study of institutional change and social representation. Health Policy Plan.

[B22] Rodriguez R, Frohlich KL (1999). The role of community organizations in the transformation of the health services delivery system in the Montreal metropolitan area. Can J Public Health.

[B23] Botha HP (1983). Primary health care according to African requirements. Isr J Med Sci.

[B24] Makoae MG, Jubber K (2008). Confidentiality or continuity? Family caregivers' experiences with care for HIV/AIDS patients in home-based care in Lesotho. Sahara J.

[B25] Government of Uganda (2005). Health Sector Strategic Plan II 2005/06 – 2009/2010.

[B26] Government of Uganda (2000). Health Sector Strategic Plan 2000/01–2004/05.

[B27] Konde-Lule J, Okuonzi S, Matsiko C, Mukanga D, Onama V, Gitta SN (2006). The Potential of the Private sector to improve health outcomes in Uganda.

[B28] Rutebemberwa E, Pariyo G, Peterson S, Tomson G, Kallander K (2009). Utilization of public or private health care providers by febrile children after user fee removal in Uganda. Malar J.

[B29] Twebaze D (2001). A Literature Review of Care-Seeing Practices for Major Childhood Illnesses in Uganda.

[B30] Patton MQ (2002). Qualitative Research & Evaluation Methods.

[B31] Dahlgren L, Emmelin M, Winkvist A (2004). Qualitative Methodology for International Public Health.

[B32] Marshall C, Rossman GB (2006). Designing Qualitative Research.

[B33] Amooti-Kaguna B, Nuwaha F (2000). Factors influencing choice of delivery sites in Rakai district of Uganda. Soc Sci Med.

[B34] Rice PL, Ezzy D (1999). Qualitative Research Methods – A Health Focus.

[B35] Dawson S, Manderson L, Tallo VL (1993). A Manual for the Use of Focus Groups.

[B36] Smith GP, Morrow HR (1996). Field trials and Health Interventions in Developing Countries: A tool box.

[B37] Khan ME, Manderson L (1992). Focus Groups in Tropical Diseases Research. Health Policy and Planning.

[B38] Holstein J, Gubrium J (1995). The Active Interview.

[B39] Graneheim UH, Lundman B (2004). Qualitative content analysis in nursing research: concepts, procedures and measures to achieve trustworthiness. Nurse Educ Today.

[B40] Clarkson MB (1995). A Stakeholder Framework for Analyzing and Evaluating Corporate Social Performance. Academy of Management Review.

[B41] Cawston PG, Mercer SW, Barbour RS (2007). Involving deprived communities in improving the quality of primary care services: does participatory action research work?. BMC Health Serv Res.

[B42] Forster R, Gabe J (2008). Voice or choice? Patient and public involvement in the National Health Service in England under New Labour. Int J Health Serv.

[B43] Gibson K (2000). The moral basis of stakeholder theory. Journal of Business Ethics.

[B44] Eldh AC, Ekman I, Ehnfors M (2008). Considering patient non-participation in health care. Health Expect.

[B45] Huezo C, Diaz S (1993). Quality of care in family planning: clients' rights and providers' needs. Adv Contracept.

[B46] World Bank (2004). World Development Report 2004: Making services work for the poor people.

[B47] Rifkin SB (1996). Paradigms lost: toward a new understanding of community participation in health programmes. Acta Trop.

[B48] Murray SA, Tapson J, Turnbull L, McCallum J, Little A (1994). Listening to local voices: adapting rapid appraisal to assess health and social needs in general practice. BMJ.

[B49] Shoo R (1991). Training primary health care workers to foster community participation. World Health Forum.

[B50] Tanner M, Lwihula GK, Burnier E, De Savigny D, Degremont A (1986). Community participation within a primary health care programme. Trop Med Parasitol.

[B51] Le PV, Jones-Le E, Bell C, Miller S (2009). Preferences for perinatal health communication of women in rural Tibet. J Obstet Gynecol Neonatal Nurs.

[B52] Berg BL (2001). Qualitative Research Methods for Social Sciences.

[B53] Flick U (1992). Triangulation Revisited: Strategy of Validation or Alternative?. Journal for the Theory of Social Behaviour.

